# Supplementary material for: A novel Chua’s based 2–D chaotic system and its performance analysis in cryptography
Source: PLoS One. 2024 Nov 6;19(11):e0306818. doi: 10.1371/journal.pone.0306818 (PMC11540234; doi:10.1371/journal.pone.0306818)
Supplement: Attachment — Submitted filename: Response-to-Reviewers.docx [file pone.0306818.s001.docx]

**Manuscript ID:** PONE-D-24-08568

**Article Title:** A Novel Chua's Based 2–D Chaotic System and Its Performance Analysis in Cryptography

We would like to extend my sincere appreciation to the reviewer and editor for their valuable time and efforts in reviewing my manuscript. Their constructive feedback and insightful comments have significantly contributed to the improvement and quality of the paper. We are truly grateful for their thorough examination and thoughtful suggestions, which have undoubtedly enhanced the overall merit of the work.

Their commitment to maintaining the standards of the journal and their dedication to fostering quality research is commendable. We are thankful for the opportunity to benefit from their expertise and guidance throughout the review process. Their contributions have been instrumental in shaping the final version of the manuscript.

Once again, thank you to the reviewer and editor for their invaluable support and for helping to strengthen the scholarly contribution of this work. We look forward to any additional insights or recommendations they may have, and We are eager to contribute further to the advancement of knowledge in our field.

**Reviewer#1, Concern # 1 (The authors have expended their time, energy and knowledge to carry out this very robust research, which is not only interesting, but also contributes immensely to existing knowledge. The work will however be more beautiful and easier to reference certain Mathematical equations, if all equations in the works are numbered rather than just numbering the 'important' model equations.):**

**Author response:** Thank you for your positive feedback and constructive suggestion. We appreciate your recognition of our efforts and the value of our research. In response to your recommendation, we will revise the manuscript to include numbering for all mathematical equations. This will enhance the clarity and ease of reference throughout the document. We believe that this adjustment will improve the overall readability and usefulness of our work for future researchers. Thank you for helping us to refine our paper.

**Author action:** We have revised the manuscript to include numbering for all mathematical equations, as per the reviewer's suggestion. This change has been implemented to improve the clarity and ease of referencing throughout the paper.

**Reviewer#2, Concern # 1 (Abstract is not clearly written as of scientific article.):**

**Author response:** Thank you for your valuable feedback regarding the clarity of the abstract. We understand the importance of a well-written abstract in conveying the scope and significance of our research effectively.

**Author action:** The abstract has been revised to improve clarity and conciseness, ensuring it accurately reflects the scope, methodology, and key findings of the study in a manner consistent with scientific article standards.

**The new abstract:**

In this study, the chaotic behavior of a second-order circuit comprising a nonlinear resistor and Chua's diode is investigated. This circuit, which includes a nonlinear capacitor and resistor among its components, is considered one of the simplest nonautonomous circuits. The research explores various oscillator characteristics, emphasizing their chaotic properties through bifurcations, Lyapunov exponents, periodicity, local Lyapunov region, and resonance. The system exhibits both a stable equilibrium point and a chaotic attractor. Additionally, the second objective of this study is to develop a novel cryptographic technique by incorporating the designed circuit into the S-box method. The evaluation results suggest that this approach is suitable for secure cryptographic applications, providing insights into constructing a cryptosystem for images and text based on its complex behavior. Real-life data are analyzed using various statistical and performance criteria after applying the proposed methodology. The findings enhance the reliability of cryptosystems. Moreover, the proposed methods are assessed using a range of statistical and performance metrics after testing on text and images. The cryptographic results are compared with existing techniques, reinforcing both the developed cryptosystem and the performance analysis of the chaotic circuit.

**Reviewer#2, Concern # 2 and Concern # 3 (Include some applicability of Chua system to improve novelty strating from general dynamical system like:**

**1. Haar wavelet approach to study the control of biological pest model in Tea plants, J Frac Calc & Nonlinear Sys (2023)4(2) : 14-30,**

**2. Nabla Generalized Fractional Riemann-Liouville Calculus On Time Scales With An Application To Dynamic Equations,** [**https://doi.org/10.48185/jfcns.v3i1.391**](https://doi.org/10.48185/jfcns.v3i1.391)**,**

**3. Study of Fractional Order Dynamical System of Viral Infection Disease under Piecewise Derivative." CMES-Computer Modeling in Engineering & Sciences 136.1 (2023).**

**4. The Volterra-Lyapunov matrix theory and nonstandard finite difference scheme to study a dynamical system." Results in Physics 52 (2023): 106890.):**

**Author response:** Thank you for your insightful suggestion regarding the inclusion of the applicability of the Chua system to improve the novelty of our work. We appreciate the examples you provided and have carefully considered your feedback.

Our research, which includes a total of 52 references, already incorporates 23 articles published between 2020 and 2023. The structure of our study is divided into three parts: the design and qualitative analysis of the circuit, the implementation of backstepping control strategies, and the application of our findings in cryptography.

In response to your suggestion, we have included the following references to enhance the applicability and relevance of our work:

1. "Study of Fractional Order Dynamical System of Viral Infection Disease under Piecewise Derivative," CMES-Computer Modeling in Engineering & Sciences 136.1 (2023).
2. "The Volterra-Lyapunov matrix theory and nonstandard finite difference scheme to study a dynamical system," Results in Physics 52 (2023): 106890.

These additions help to contextualize the Chua system within broader applications and underline the novel aspects of our research.

**Author action:** We have revised the manuscript to include the suggested references, demonstrating the broader applicability of the Chua system. This enhancement aligns our work with contemporary research trends and highlights the innovative aspects of our study.

**Reviewer#2, Concern # 4 (There** **are many mistake related to grammer correct.):**

**Author response:** Thank you for your feedback regarding grammatical errors in the manuscript. We understand the importance of clear and accurate language in effectively communicating our research.

**Author action:** We have utilized an AI tool, specifically Paperpal Copilot, to thoroughly review and correct grammatical errors throughout the manuscript. This ensures that the language is clear and adheres to high standards of academic writing.

**Reviewer #2, Concern # 5 and Concern # 7 (Slightly discuss existence of such system mathematically using the idea:**

**On complex fractal-fractional order mathematical modeling of CO_2_ emanations from energy sector." Physica Scripta 99.1 (2023): 015226.**

**Check references some items are missing):**

**Author response:** Thank you for your insightful feedback and for suggesting the reference on complex fractal-fractional order mathematical modeling. While our research is primarily focused on differential equations, your suggestion has opened new avenues for considering fractional order modeling for this system.

**Author action:** We acknowledge the importance of fractional order modeling and are currently working on the numerical simulation part of this aspect, which we plan to include in our future work. Meanwhile, we have added the suggested reference,

1. "On complex fractal-fractional order mathematical modeling of CO2 emanations from energy sector," Physica Scripta 99.1 (2023): 015226, to our reference list.

Additionally, we have reviewed and corrected any missing items in the references to ensure completeness and accuracy.

**Reviewer#2, Concern # 6 (Revise conclusion):**

**Author response:** Thank you for your suggestion to revise the conclusion of our manuscript. We appreciate your feedback and have carefully considered your input.

**Author action:** Following your suggestion, we have modified the conclusion section of the manuscript to better encapsulate the key findings and implications of our research. The revised conclusion provides a more comprehensive summary of our study's contributions and highlights the significance of our findings in the broader context of the field. We believe that these changes enhance the overall clarity and impact of our paper.

**Revised Conclusion:**

This research involves constructing a circuit comprising a capacitor, resistor, inductor, and Chua's circuit. Utilizing existing models for the inductor, capacitor, and resistor, this study applies Kirchhoff’s voltage-current law to derive the dynamic equations for the system. The dynamic properties of a novel chaotic oscillator were investigated, demonstrating its chaotic nature. Techniques such as bifurcation analysis, Lyapunov exponents, Poincaré maps, local Lyapunov exponent regions, periodicity, and resonance were employed to analyze the oscillator, while classical dynamics were used to examine the stability of the designed circuit.

The analysis of Lyapunov exponents and bifurcation diagrams reveals that the chaotic system exhibits intricate dynamical behavior as the initial conditions and parameters change. Additionally, research on these systems has uncovered unique phenomena, including state transitions and the coexistence of attractors. Its diverse dynamic properties demonstrate its applicability to chaotic text and image cryptosystems.

The effectiveness of the proposed mechanisms was assessed using various methods, with results highlighting critical characteristics essential for reliable security purposes. Furthermore, a novel text and image cryptosystem is presented, utilizing the proposed chaotic sequence method along with the S-box approach. The efficiency of the recommended mechanisms has been evaluated through various assessments, indicating the essential qualities for ensuring trustworthy security. Moreover, the proposed encryption and decryption algorithms' outputs are compared with existing results, further reinforcing the effectiveness and robustness of the proposed chaotic oscillator circuit.
